# Promoting patient participation in the cancer consultation: evaluation of a prompt sheet and coaching in question-asking

**DOI:** 10.1038/sj.bjc.6690346

**Published:** 1999-04

**Authors:** R Brown, P N Butow, M J Boyer, M H N Tattersall

**Affiliations:** Medical Psychology Unit, Blackburn Building, Department of Cancer Medicine, University of Sydney, Camperdown, NSW 2006, Australia; Department of Medical Oncology, Royal Prince Alfred Hospital, Camperdown, NSW 2006, Australia

**Keywords:** interventions, patient participation, question prompt sheet

## Abstract

Active participation in the medical consultation has been demonstrated to benefit aspects of patients' subsequent psychological well-being. We investigated two interventions promoting patient question-asking behaviour. The first was a question prompt sheet provided before the consultation, which was endorsed and worked through by the clinician. The second was a face to face coaching session exploring the benefits of, and barriers to, question-asking, followed by coaching in question-asking behaviour employing rehearsal techniques. Sixty patients with heterogeneous cancers, seeing two medical oncologists for the first time, were randomly assigned to one of three groups: two intervention groups and one control group. Sociodemographic variables and anxiety were assessed prior to the intervention which preceded the consultation. The consultations were audiotaped and subsequently analysed for question-asking behaviour. Anxiety was assessed again immediately following the consultation. Questionnaires to assess patient satisfaction, anxiety and psychological adjustment were sent by mail 2 weeks following the consultation. Presentation and discussion of the prompt sheet significantly increased the total number of questions asked and the number of questions asked regarding tests and treatment. Coaching did not add significantly to the effects of the prompt sheet. Psychological outcomes were not different among the groups. We conclude that a question prompt sheet addressed by the doctor is a simple, inexpensive and effective means of promoting patient question asking in the cancer consultation. © 1999 Cancer Research Campaign


*British Journal of Cancer* (1999) **80**(1/2), 242–248
© 1999 Cancer Research Campaign
Article no. bjoc.1998.0346

### Promoting patient participation in the cancer consultation: evaluation of a prompt sheet and coaching in question-asking

**R Brown[1], PN Butow[1], MJ Boyer[3] and MHN Tattersall[2]**

[1]Medical Psychology Unit, Blackburn Building, [2]Department of Cancer Medicine, University of Sydney, Camperdown, NSW 2006, Australia; [3]Department of Medical Oncology, Royal Prince Alfred Hospital, Camperdown, NSW 2006, Australia

**Summary** Active participation in the medical consultation has been demonstrated to benefit aspects of patients' subsequent psychological well-being. We investigated two interventions promoting patient question-asking behaviour. The first was a question prompt sheet provided before the consultation, which was endorsed and worked through by the clinician. The second was a face to face coaching session exploring the benefits of, and barriers to, question-asking, followed by coaching in question-asking behaviour employing rehearsal techniques. Sixty patients with heterogeneous cancers, seeing two medical oncologists for the first time, were randomly assigned to one of three groups: two intervention groups and one control group. Sociodemographic variables and anxiety were assessed prior to the intervention which preceded the consultation. The consultations were audiotaped and subsequently analysed for question-asking behaviour. Anxiety was assessed again immediately following the consultation. Questionnaires to assess patient satisfaction, anxiety and psychological adjustment were sent by mail 2 weeks following the consultation. Presentation and discussion of the prompt sheet significantly increased the total number of questions asked and the number of questions asked regarding tests and treatment. Coaching did not add significantly to the effects of the prompt sheet. Psychological outcomes were not different among the groups. We conclude that a question prompt sheet addressed by the doctor is a simple, inexpensive and effective means of promoting patient question asking in the cancer consultation.

**Keywords:** interventions; patient participation; question prompt sheet

An examination of trends in clinician behaviour in Western cultures over 3 decades reveals a move away from a paternalistic style, characterized by providing minimal information to patients (Oken, 1961). Previously, doctors usually withheld detailed information regarding cancer diagnosis, prognosis and treatment options in the belief that such information would cause the patient excessive fear, anxiety and loss of hope, thus worsening patient outcomes (Oken, 1961; Mosconi et al, 1991; Girgis and Sanson-Fisher, 1995). In a 1961 study of 219 physicians from a range of specialties in the USA, Oken (1961) reported that 90% indicated a preference for not informing patients about their cancer diagnosis. A 1979 replication of the Oken (1961) study revealed that 98% of 264 physicians affirmed the value of providing detailed information to cancer patients, and 100% endorsed the patient's right to be informed of the diagnosis (Novack et al, 1979). Various reasons have been offered to account for this shift in clinician behaviour, including: (1) an increased fear of litigation among physicians, (2) the publication of guidelines for the disclosure of diagnoses (Reiser, 1980), (3) more effective therapies for cancer patients through technological advancement and (4) a change in public opinion regarding patients' rights to medical information and full disclosure (Thomasma, 1983).

Western cancer patients who are informed of their diagnosis are increasingly unwilling to adopt a traditional, passive role in the medical consultation. Patients commonly now seek information to enable them to make decisions about treatment, to understand prognostic issues and to be clear about treatment side-effects (Roter, 1977; Tattersall et al, 1994). Patient advocacy groups have endorsed a consumeristic philosophy, which portrays the physician as a service provider. Self-help groups have been established which provide support and training for patients and their carers, who also often pursue a more active role in the medical consultation (Levin et al, 1976; Green et al, 1977; Cassileth et al, 1980; Tattersall et al, 1994).

Patients who actively participate are able to change the focus of the consultation and influence the duration and the amount of information provided (Kaplan et al, 1996). Research has demonstrated a relationship between the active pursuit of information and involvement in treatment decisions, and improved psychological adjustment and increased patient satisfaction (Korsch et al, 1968; Kupst et al, 1975; Bertakis et al, 1991; Butow et al, 1994). The direction of causality, however, is unclear. Patient activity may cause such beneficial outcomes or, alternatively, well-adjusted patients who are satisfied with their care may ask more questions.

While patients apparently benefit from active participation, evidence shows that some clinicians still dominate the consultation process. In a recent Australian study, a cancer-specific computerized interaction analysis system was used to investigate the relationship between physician–patient behaviour and patient outcomes in the initial medical oncology consultations of 142 cancer patients (Butow et al, 1995). Patients spoke for 24% of the total consultation time and asked, on average, 5.6 questions that took up 0.07% of this time. In contrast, the physician spoke for 44% of the consultation time, and only 5% of this was spent

*Received 19 May 1998*
*Revised 16 October 1998*
*Accepted 4 November 1998*

*Correspondence to:* R Brown answering patient questions. Roter (1977), who examined the question-asking behaviour of 250 general practice patients, obtained similar results in an intervention study. In this study the control group asked an average of 1.2 questions. These results show that patients ask few questions during the consultation and that physicians are dominating the verbal exchange.

Patients may not be asking questions for a variety of reasons. These reasons include, (a) an unwillingness to appear ignorant, (b) lack of knowledge about the illness and not knowing which questions to ask, (c) patient belief in an expert authority and, (d) unease about communication with a person of a perceived higher status (Roter, 1977). Conversely, some physicians may feel uncomfortable answering patients' questions because it reduces their control over the consultation.

Roter (1977) suggests that patients' question-asking behaviour can be influenced by addressing three contributing elements namely: (1) enabling, (2) predisposing and (3) reinforcing factors. Applying this model, Roter (1977) employed an individual coaching intervention in which patients were encouraged to ask questions of the general practitioner. This intervention, while successful in increasing patients' question-asking behaviour, required considerable resources and increased the negative interchanges between the doctor and patient.

To overcome some of these structural difficulties, Butow et al (1994) investigated, in a randomized trial, the presentation of a question prompt sheet (QPS) designed to encourage patient question-asking behaviour in cancer consultations. Results showed that the overall number of questions did not vary significantly between groups; however, patients given the prompt sheet asked significantly more questions regarding their prognosis.

The studies by Roter (1977) and Butow et al (1994) demonstrate the limited efficacy of their interventions in increasing question-

**Table 1** Demographic and disease characteristics of sample ($n = 60$)

| | **Control group ($n = 20$)** | **QPS group ($n = 20$)** | **QPS + coach group ($n = 20$)** | **Total sample ($n = 60$)** |
|---|---|---|---|---|
| Age | | | | |
| Mean | 53.1 years | 52.8 years | 52.9 years | 53 years |
| Range | 32–71 | 17–71 | 29–77 | 17–77 |
| Gender | | | | |
| Female | 11 (55%) | 9 (45%) | 11 (55%) | 31 (52%) |
| Male | 9 (45%) | 11 (55%) | 9 (45%) | 29 (49%) |
| Education level | | | | |
| Completed ≤ 10 years High School | 11 (45%) | 10 (50%) | 12 (60%) | 33 (55%) |
| Completed High School (12 years) | 2 (10%) | 1 (5%) | 3 (15%) | 6 (10%) |
| Tertiary Non-University | 3 (15%) | 1 (5%) | 2 (10%) | 6 (10%) |
| | 4 (20%) | 8 (40%) | 3 (15%) | 15 (25%) |
| Tertiary University | | | | |
| Occupation | | | | |
| Professionals | 11 (55%) | 12 (60%) | 9 (45%) | 32 (53%) |
| Trades people | 2 (10%) | 0 | 5 (25%) | 7 (12%) |
| Clerks and sales | 3 (15%) | 3 (15%) | 3 (15%) | 9 (15%) |
| Labourers | 2 (10%) | 4 (20%) | 2 (10%) | 8 (13%) |
| Home duties/students | 2 (10%) | 1 (5%) | 1 (5%) | 4 (7%) |
| Marital status | | | | |
| Single | 2 (10%) | 3 (15%) | 3 (15%) | 8 (12%) |
| Married | 15 (75%) | 12 (60%) | 15 (75%) | 42 (70%) |
| Divorced/Separated | 2 (10%) | 3 (15%) | 1 (5%) | 6 (10%) |
| Common Law | 0 | 1 (5%) | 1 (5%) | 2 (3%) |
| Widowed | 1 (5%) | 1 (5%) | 0 | 2 (3%) |
| Type of cancer | | | | |
| Breast | 6 (30%) | 5 (25%) | 5 (25%) | 16 (27%) |
| Lung | 2 (10%) | 4 (20%) | 1 (5%) | 7 (12%) |
| Testes | 1 (5%) | 3 (15%) | 0 | 4 (7%) |
| Prostate | 4 (20%) | 1 (5%) | 2 (10%) | 7 (12%) |
| Colorectal | 0 | 1 (5%) | 5 (25%) | 6 (10%) |
| Other | 7 (35%) | 6 (30%) | 7 (35%) | 20 (33%) |
| Stage | | | | |
| Loco-regional | 11 (55%) | 11 (55%) | 7 (35%) | 29 (48%) |
| Metastasis | 9 (45%) | 9 (45%) | 13 (65%) | 31 (52%) |
| Estimated prognosis | | | | |
| ≤ 1 year | 8 (40%) | 8 (40%) | 6 (30%) | 22 (37%) |
| 1–5 years | 9 (45%) | 7 (35%) | 10 (50%) | 26 (43%) |
| Normal life expectancy | 3 (15%) | 5 (25%) | 4 (20%) | 12 (20%) |
| Time since diagnosis | | | | |
| ≤2 months | 11 (55%) | 11 (55%) | 13 (65%) | 35 (58%) |
| >2 months | 9 (45%) | 9 (45%) | 7 (35%) | 25 (42%) |

*© Cancer Research Campaign 1999*

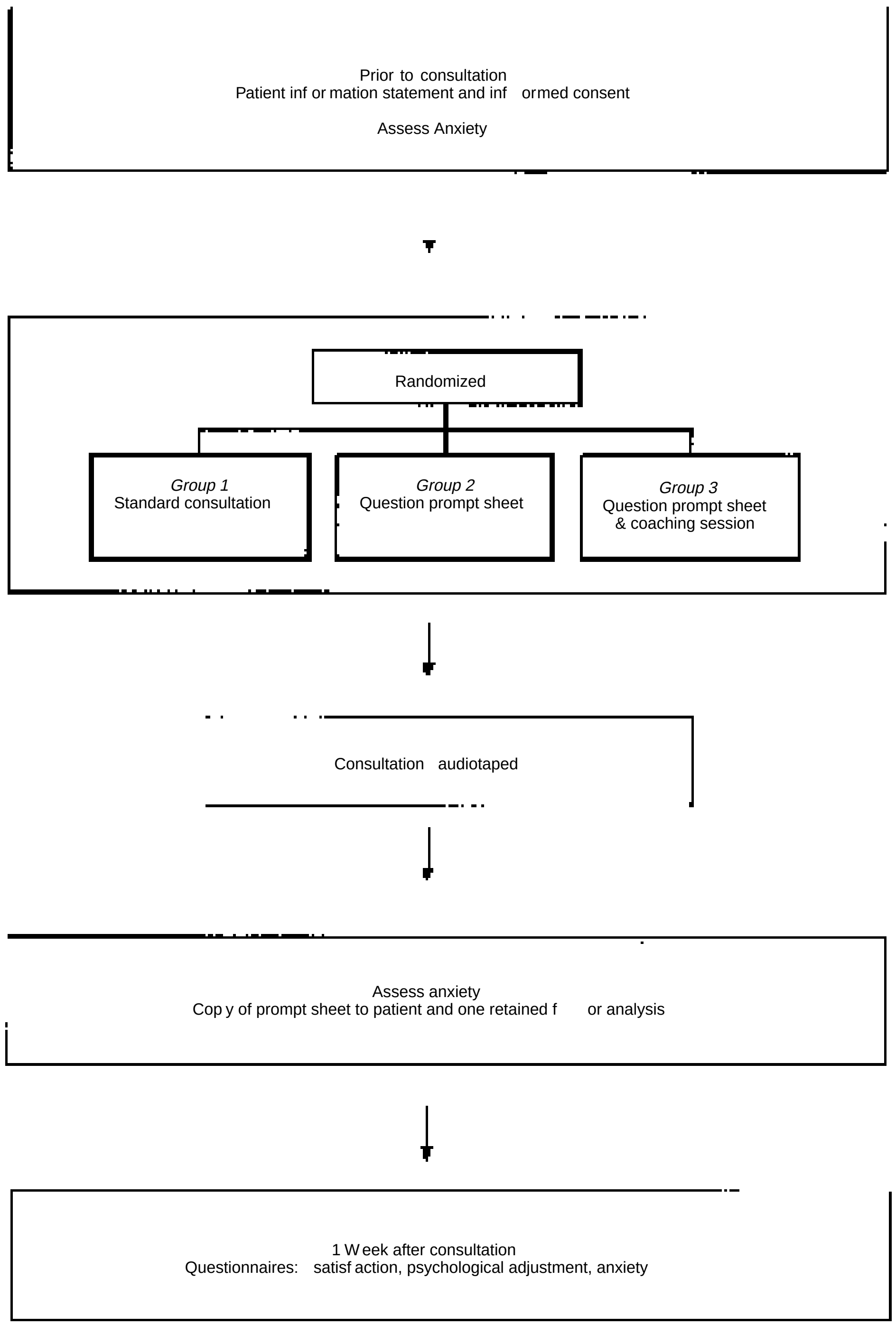

**Figure 1** Experimental protocol asking by patients. However, both studies have limitations: (a) the physician was not involved in the intervention process in either study, (b) Roter's study was conducted in a general practice and the generalizability to the cancer setting is questionable, and (c) the additive effects of a question prompt sheet and individual coaching were not explored.

The current study investigated the effects of providing a question prompt sheet that the physician endorsed and discussed, and the added effect of an individualized method of coaching patients in question-asking behaviour. The aim of this study was to: (a) evaluate two interventions designed primarily to influence physician–patient communication and to increase question asking

*© Cancer Research Campaign 1999*

behaviour and (b) to investigate the impact of increased question asking on psychological outcome measures.

### MATERIALS AND METHODS

Consecutive patients seeing two medical oncologists for the first time at a tertiary referral teaching hospital in Sydney, Australia, were invited to participate in the study. No patients refused to participate at the initial invitation. Following the consultation, one patient, who was seeking a second opinion and preferred to continue treatment with the original oncologist, refused a tape copy and further involvement in the study. Thirty-one females and 29 males, with an average age of 53.1 years were recruited. Forty-five per cent of patients had completed secondary school education, university or some other form of tertiary training, while 55% had completed 10 years school education. These rather elevated levels of education were matched by correspondingly high numbers of patients in the managerial or professional occupation groups (53%). The sample was heterogeneous for primary site of the cancer with 26.6% having a diagnosis of breast cancer, 11.6% lung, 11.6% prostate and the remainder distributed evenly across sites (Table 1).

Patients were informed of the purpose and the requirements of the study and permission was sought to audiotape the consultation. Eligible patients were randomly allocated to one of three groups of equal size. Group 1 – Standard practice consultation. Group 2 – Participants were provided with a question prompt sheet (QPS) which contained a structured list of 17 questions commonly asked by patients of their medical oncologist. The questions were derived from a content analysis of 20 taped consultations and consultation with four experts: two medical oncologists and two psychologists experienced in cancer research. These questions were grouped according to their content using a method of categorization described by Ley et al (1973). The categories include questions concerning diagnosis, tests, treatment, prognosis, psychosocial issues and support services available (Figure 2). The doctor endorsed the prompt sheet and, towards the end of the consultation, went through each category eliciting and answering questions according to a standard protocol. Group 3 – Participants received the QPS plus an interactive coaching session immediately before the consultation, with a research psychologist. The coaching covered the following areas:

*Question generation:* The importance of asking questions during the consultation was discussed and patients were asked to generate a list of questions for the doctor. These questions were compared with those on the QPS and new questions were added to the sheet.

*Exploration of benefits and barriers:* Benefits of, and barriers to, asking questions were elicited.

*Rehearsal:* Participants were invited to imagine a situation in which they would experience difficulty asking questions of the doctor. Participants were asked to imagine themselves in this situation and then to rehearse asking questions using the researcher as a surrogate doctor.

Prior to the consultation and the introduction of the interventions, all participants completed a short questionnaire measuring anxiety. The consultations were audiotaped to allow analysis of information presented in the consultation and collation of the number of questions asked by patients. One of us listened to each audiotape and counted patient questions within each category of the QPS. The category subtotals were summed to calculate a total number of questions asked by each patient.

Immediately following the consultation, anxiety was assessed. Seven to 10 days following the consultation, participants were mailed questionnaires to assess satisfaction with the consultation, anxiety and psychological adjustment to cancer. Anxiety was measured using the Spielberger State Anxiety Scale (SSAS) (Spielberger, 1983) which is a widely used scale measuring situational anxiety. Patient satisfaction with the consultation was assessed during the follow-up phase using a 25-item Likert scale adapted from Roter (1977) and Korsch et al (1968). This scale assessed satisfaction with: (1) the amount and quality of information presented, (2) the communication skills demonstrated by the doctor and (3) the level of patient participation in the consultation. The internal reliability of this scale in a sample of 80 patients

**How to make the most of your time with the doctor**

Most people who see their doctor for the first time have questions and concerns. Often these get forgotten in the rush of the moment, only to be remembered later. To help you make the most of your time with the doctor we have compiled a list of questions people often ask. We suggest that you tick those you want to ask and then write down any other specific questions you have in the space provided.

You can keep this sheet with you when you see the doctor. You may find that the doctor answers your questions without you even asking, but this sheet can serve as a checklist so that you know that you have covered everything that is important to you.

*Questions people often ask*

1 What kind of cancer do/did I have?
2 Where is the cancer at the moment? Has it spread?
3 What symptoms will the cancer cause?
4 Will I need any more tests?
5 If so, will they hurt?
6 What will they tell us?
7 What treatment will I need?
8 Does the treatment have any side-effects? If so, what can be done about them?
9 What should I do or not do while having treatment?
10 How long will it be before I know if the treatment is working?
11 Will my family be affected by my cancer?
12 Will my work be affected?
13 Will my sexual life be affected?
14 What will the outcome be? Will I get better?
15 If we get rid of the cancer, what are the chances of it coming back?
16 Do members of my family have a greater risk of getting cancer?
17 Are there services available to help me cope with this illness?

Write any other questions you have in the space below:

..................................................................................................

..................................................................................................

..................................................................................................

..................................................................................................

**Figure 2** Question prompt sheet

© Cancer Research Campaign 1999

enrolled in a similar study was high (Cronbach's Alpha = 0.91). Psychological adjustment to the diagnosis of cancer was measured using the Mental Adjustment to Cancer Scale (MAC) (Watson et al, 1988). This scale has been used in previous studies and has shown good reliability and sensitivity.

*Sample size:* In a previous study (Butow et al, 1994), patients asked a mean number of 5.5 (s.d. 4.0) questions. A sample size of 20 per group would enable a difference in the mean number of questions asked as small as four to be detected with a power of 0.80 and a significance level of 0.05.

*Statistics:* Kruskall–Wallis one-way analysis of variances (ANOVAs) were used to detect differences in question asking between the three experimental groups while Mann–Whitney *U*-tests were used to detect differences between the combined intervention groups and the control group. Differences in satisfaction between the intervention group and the control group were measured using Mann–Whitney *U*-tests. Independent samples *t*-tests were used to explore differences between groups on both the anxiety and psychological adjustment measures.

### RESULTS

An exploration of demographic and disease variables between the three experimental groups revealed no major imbalances (Table 1).

### Impact of prompt sheet and coaching on question-asking

Patients in the control group asked a median of 8.5 questions (IQR, 5–21.5), the prompt sheet group 15 (IQR, 10.7–26.7) and the coaching group 13 (IQR, 8–27.7). A Kruskall–Wallis one-way ANOVA was conducted to examine differences among the three groups in the total number of questions asked. The result was not significant; however, there was a tendency for patients in either intervention group to ask more questions than those in the control group.

As Butow et al (1994) found that a QPS increased question-asking in one question category (prognosis), an analysis of the total number of questions asked in each of the question categories according to the three groups was conducted. The results were non-significant in any category.

As there was no significant difference in the number of questions asked between participants receiving a QPS (median = 15; IQR, 10.75–26.75) and those who received coaching in addition to the prompt sheet (median = 13; IQR, 8.0–27.75, $P = 0.65$), the two groups were combined. All subsequent analysis was conducted comparing the control group with the combined intervention groups that had received a prompt sheet. Patients in the intervention group asked a median number of 14 questions (IQR, 8.2–26.7). A Mann–Whitney *U*-test was used to assess the effect of the prompt sheet on total question-asking. The result indicated that the prompt sheet increased question-asking significantly ($z = -2.025$, $P = 0.043$).

An analysis of the number of questions asked in each of the question categories, according to the provision of a prompt sheet or not, was then conducted. The result demonstrated that patients with a prompt sheet asked significantly more questions (median = 1, IQR, 0–3) than patients in the control group (median = 0, IQR, 0–1.75) regarding tests ($z = -1.974$, $P = 0.048$). Differences between the groups in questions asked about other topics were not statistically significant (Table 2).

**Table 2** Median number and range of questions asked according to categories between the control and prompt sheet groups

| | Control group (*n* = 20) | | Combined QPS + coach groups (*n* = 40) | | |
|---|---|---|---|---|---|
| | Median | IQR | Median | IQR | *P*-value |
| Diagnosis | 1.0 | 0.0–4.8 | 1.0 | 0.0–4.0 | 0.6128 |
| Tests | 0.0 | 0.0–1.8 | 1.0 | 0.0–3.0 | 0.0483[a] |
| Treatment | 4.0 | 0.5–10.8 | 7.0 | 3.0–10.7 | 0.2442 |
| Prognosis | 0.0 | 0.0–1.8 | 1.0 | 0.0–2.8 | 0.1478 |
| Other medical | 0.0 | 0.0–1.8 | 1.0 | 0.0–3.0 | 0.2059 |
| Psychosocial | 0.0 | 0.0–1.0 | 1.0 | 0.0–3.0 | 0.1119 |
| Help available | 0.0 | 0.0–0.0 | 0.0 | 0.0–0.0 | 0.1049 |
| Total | 8.5 | 5.0–21.5 | 14 | 8.3–26.8 | 0.0429* |

[a]Significant at 0.05.

On the assumption that the interventions may have prompted non-active patients to ask at least one question, rather than increasing the total number of questions asked, the patients were grouped according to whether they had asked no questions, or one or more questions. In this analysis, the treatment category $\chi^2$ was significant ($\chi^2_1 = 0.295$, $P = 0.024$), while in other topic areas results were non-significant. There was also a tendency for more patients in the intervention groups 27/40 (67.5%) than those in the control group 9/20 (45%), to ask at least one question about prognosis ($\chi^2_{1.} = 2.8$, $P = 0.09$).

### Impact of prompt sheet on anxiety, satisfaction and psychological adjustment to cancer

#### *Anxiety*

Independent samples *t*-tests were used to assess differences in anxiety between the combined prompt sheet group and the control group before and after the consultation. The results were non-significant, indicating that anxiety was not affected by the use of a prompt sheet. To assess differences among groups on the extent to which anxiety changed following the consultation, change scores were computed between the immediate pre-consultation and immediate post-consultation measurements. Independent samples *t*-tests conducted on these scores to investigate differences among groups were non-significant (Table 3). Kruskall–Wallis one-way ANOVAs used to assess the relationship between question-asking and anxiety scores, both immediately following the consultation and 1 week post-consultation, were non-significant.

#### *Satisfaction*

While the scores on the satisfaction scale exhibited considerable variability (52–124) the distribution was significantly skewed with most people reporting high satisfaction with the consultation. A Mann–Whitney *U*-test used to explore differences in satisfaction between the combined intervention group and the control group was non-significant, indicating that increased question-asking was not associated with total satisfaction with the consultation (Table 3).

#### *Psychological adjustment*

The Mental Adjustment to Cancer (MAC) (Watson et al, 1988) scale yields four subscales: fighting spirit, helpless/hopeless, anxiety and fatalism. Two of the subscales were used for this

 © Cancer Research Campaign 1999

**Table 3** Mean scores according to psychosocial measures between the control and prompt sheet groups.

| | Control group ($n$ = 20) | | Combined QPS + coach groups ($n$ = 40) | | |
|---|---|---|---|---|---|
| | **Mean** | **s.d.** | **Mean** | **s.d.** | ***P*-value** |
| Anxiety | | | | | |
| Pre-consultation | 46.4 | 5.5 | 47.0 | 7.5 | 0.772 |
| Immediate post-consultation | 48.5 | 6.1 | 47.2 | 7.9 | 0.540 |
| Change scores | 1.947 | 5.892 | 0.250 | 7.7476 | 0.389 |
| Psychological adjustment (MAC subscales) | | | | | |
| Fighting spirit | 50.0 | 5.1 | 49.7 | 5.5 | 0.882 |
| Helpless/hopeless | 9.5 | 3.3 | 9.9 | 2.9 | 0.658 |
| Satisfaction | Median | IQR | Median | IQR | P-value |
| | 108.0 | 100–109 | 107 | 97–113.5 | 0.705 |

analysis. The fighting spirit factor was used since it encompassed positive aspects of psychological adjustment to the diagnosis. The helpless/hopeless subscale was also analysed to detect whether the use of the prompt sheet was associated with an improvement in patients' sense of control over the illness as a consequence of information provision. As these variables were normally distributed, two independent samples *t*-tests were used to explore differences in psychological adjustment between the control and the prompt sheet. The *t*-tests produced non-significant results indicating that the prompt sheet did not significantly affect fighting spirit or the patients' sense of helplessness/hopelessness (Table 3).

### DISCUSSION

We evaluated two interventions designed to increase question-asking and to test the assertions stated in the theoretical and practical literature concerning the benefits of active patient participation in the medical consultation. Our results indicate that patients who were provided with a prompt sheet asked significantly more questions in general than those without and, in particular, asked more questions about tests and treatment. The addition of a complex coaching intervention to the simpler prompt sheet did not increase further the number of questions asked. Our results support those of Roter (1977) and Butow et al (1994). However, Butow found that the use of a prompt sheet raised question-asking only in the prognosis category and not overall. In our sample, far more patients in the combined intervention group (67.5%) asked at least one question regarding prognosis than patients in the control group (45%); however, while this result was in the expected direction, the result of the $\chi^2$ was not statistically significant. These percentages are considerably higher than the percentages reported by Butow et al (1994) (35% for the prompt sheet group and 16% for the control).

A comparison of the mean number of total questions asked between the intervention groups in the Butow et al (1994) and the Roter (1977) studies (5.6 and 2.2 respectively), and the intervention groups in the current study (16.3), reveals that patients in the current sample asked far more questions overall than patients in the previous studies. The current patients were more highly educated and in more prestigious occupations than the general population, which may account for an increase in the number of questions asked. However, it is also possible that the endorsement of the prompt sheet by the clinician and discussion of the selected questions encouraged patients to ask a greater number of questions than previous samples.

The fact that significant differences were detected in the number of questions asked about tests and treatment in particular, suggests that patients benefit most from additional encouragement to explore these areas.

### The failure of the coaching intervention

The coaching intervention addressed the model proposed by Roter (1977); however, discussion of the benefits of, and barriers to, question-asking did not translate to increased question-asking, or to increase the patient's sense of control over the consultation. The role-playing technique, based on rehearsal of a behaviour prior to a stressful event, did not cause patients to ask more questions in the consultation. As the differences between groups were so small it is unlikely that insufficient power is to blame for this result. Perhaps a ceiling in the desired question-asking had been achieved by the doctor-endorsed question prompt sheet. Alternatively, an intervention such as this may need to be conducted on more than one occasion in order to produce a substantial change in assertive behaviour.

### Psychological outcomes

Patients' anxiety levels did not change measurably as a result of the provision of the prompt sheet, indicating that this intervention can be used by patients without increasing their distress. Anxiety also remained stable over the trial period in all three groups. This result is at odds with the literature, which suggests that providing information and involving the patient in the consultation reduces anxiety (Derogatis et al, 1983; Molleman et al, 1984). However, anxiety levels may have been stable in this sample, as approximately 40% of patients had known of their diagnosis for 3 or more months prior to the study consultation. Similarly, perhaps psychological adjustment to the diagnosis and treatment of cancer is influenced by such a complex array of factors that short-term interventions, such as those employed in the present study, would not have impact.

Satisfaction with the consultation was similarly unaffected by the interventions studied. Satisfaction questionnaires have been demonstrated to be insensitive in detecting dissatisfaction and

*© Cancer Research Campaign 1999* 

many studies employing satisfaction as an outcome measure report a positive skew (Kupst et al, 1975; Blanchard et al, 1990; Brown et al, 1997). This study is no exception. Perhaps cancer patients overlook characteristics of the oncologist or features of the consultation with which they are dissatisfied more than general practice patients with less severe illnesses, as cancer patients must rely heavily on the knowledge and skills of their oncologist and need to believe that their oncologist is capable.

In conclusion, this study showed that whilst a question prompt sheet addressed by the physician significantly increased question-asking, the addition of one-to-one intensive coaching with a psychologist before an initial oncology consultation did not further enhance question-asking. A prompt sheet that the physician endorses is an effective and inexpensive means of encouraging patient involvement in the cancer consultation.

### REFERENCES

Bertakis KD, Roter D and Putnam SM (1991) The relationship of physician medical interview style to patient satisfaction. *The Journal of Family Practice* **32**: 175–181

Blanchard CG, Labreque BA, Ruckdeschel JC and Blanchard EB (1990) Physician behaviours, patient perceptions, and patient characteristics as predictors of satisfaction of hospitalized adult cancer patients. *Cancer* **65**: 186–192

Brown RF, Dunn S and Butow PN (1997) Meeting patient expectation in the cancer consultation. *Ann Oncol* **8**: 1–5

Butow PN, Dunn SM, Tattersall MHN and Jones QJ (1994) Patient participation in the cancer consultation: evaluation of a question prompt sheet. *Ann Oncol* **5**: 199–204

Butow PN, Dunn SM, Tattersall MHN and Jones QJ (1995) Computer based interaction analysis of the cancer consultation. *Br J Cancer* **71**: 1115–1121

Cassileth BR, Zupkis RV, Sutton-Smith K and March V (1980) Information and participation preferences among cancer patients. *Ann Int Med* **92**: 832–836

Derogatis LR, Morrow GR and Fetting J (1983) The prevalence of psychiatric disorders among cancer patients. *JAMA* **249**: 751–757

Girgis A and Sanson-Fisher RW (1995) Breaking bad news: consensus guidelines for medical practitioners. *J Clin Oncol* **13**: 2449–2456

Green LW, Werlin SH and Schauffler HH (1977) Research and demonstration issues in self care: measuring the decline of medicocentrism. *Health Education Monogr* **5**: 161–189

Kaplan SH, Greenfield S, Gandek B, Rogers WH and Ware JE (1996) Characteristics of physicians with participatory decision-making styles. *Ann Int Med* **124**: 497–504

Korsch BM, Gozzi EK and Francis V (1968) Gaps in doctor–patient communication: doctor patient interaction and patient satisfaction. *Pediatrics* **42**: 855–870

Kupst M, Dresser K, Schulman JL and Paul MH (1975) Evaluation of methods to improve communication in the physician–patient relationship. *Am J Orthopsychiatry* **45**: 420–429

Levin L, Katz AH and Holst E (1976) *Lay Initiatives in Health*. Prodist: New York

Ley P, Bradshaw PW, Eaves D and Walker CM (1973) A method for increasing patients recall of information presented by doctors. *Psychol Med* **3**: 217–220

Molleman E, Krabbendam PJ, Annyas AA, Koops HS, Sleijfer DT and Vermey A (1984) The significance of the doctor–patient relationship in coping with cancer. *Soc Sci Med* **18**: 475–480

Mosconi P, Meyerowitz BE, Liberati MC and Liberati A (1991) Disclosure of breast cancer diagnosis: patients and physician reports. *Ann Oncol* **2**: 273–280

Novack DH, Freireich EJ and Vaisrub S (1979) Changes in physicians attitudes toward telling the cancer patient. *JAMA* **241**: 897–900

Oken D (1961) What to tell cancer patients: a study of medical attitudes. *JAMA* **175**: 1120–1128

Reiser SJ (1980) Words as scalpels: transmitting evidence in the clinical dialogue. *Ann Int Med* **92**: 837–842

Roter DL (1977) Patient participation in the patient provider interaction: the effects of patient question asking on the quality of interaction, satisfaction and compliance. *Health Education Monogr* **5**: 281–315

Spielberger CD (1983) *Manual for the State Trait Anxiety Inventory (Form Y)*. Consulting Psychologists Press: Palo Alto, CA

Tattersall MHN, Butow PN, Griffin AM and Dunn SM (1994) The take home message: patients prefer consultation audiotapes to summary letters. *J Clin Oncol* **12**: 1305–1311

Thomasma DC (1983) Beyond medical paternalism and patient autonomy: a model of physician conscience for the physician–patient relationship. *Ann Int Med* **98**: 243–248

Waston M, Greer S, Young J, Inayat Q, Burgess C and Robertson B (1988) Development of a questionnaire measure of adjustment to cancer: the MAC scale. *Psychol Med* **18**: 203–209

 *© Cancer Research Campaign 1999*

## References

[PDF_00535] Bertakis K. D., Roter D., Putnam S. M. (1991). The relationship of physician medical interview style to patient satisfaction.. J Fam Pract.

[PDF_00538] Blanchard C. G., Labrecque M. S., Ruckdeschel J. C., Blanchard E. B. (1990). Physician behaviors, patient perceptions, and patient characteristics as predictors of satisfaction of hospitalized adult cancer patients.. Cancer.

[PDF_00546] Butow P. N., Dunn S. M., Tattersall M. H., Jones Q. J. (1995). Computer-based interaction analysis of the cancer consultation.. Br J Cancer.

[PDF_00543] Butow P. N., Dunn S. M., Tattersall M. H., Jones Q. J. (1994). Patient participation in the cancer consultation: evaluation of a question prompt sheet.. Ann Oncol.

[PDF_00548] Cassileth B. R., Zupkis R. V., Sutton-Smith K., March V. (1980). Information and participation preferences among cancer patients.. Ann Intern Med.

[PDF_00550] Derogatis L. R., Morrow G. R., Fetting J., Penman D., Piasetsky S., Schmale A. M., Henrichs M., Carnicke C. L. (1983). The prevalence of psychiatric disorders among cancer patients.. JAMA.

[PDF_00552] Girgis A., Sanson-Fisher R. W. (1995). Breaking bad news: consensus guidelines for medical practitioners.. J Clin Oncol.

[PDF_00554] Green L. W., Werlin S. H., Schauffler H. H., Avery C. H. (1977). Research and demonstration issues in self-care: measuring the decline of medicocentrism.. Health Educ Monogr.

[PDF_00557] Kaplan S. H., Greenfield S., Gandek B., Rogers W. H., Ware J. E. (1996). Characteristics of physicians with participatory decision-making styles.. Ann Intern Med.

[PDF_00560] Korsch B. M., Gozzi E. K., Francis V. (1968). Gaps in doctor-patient communication. 1. Doctor-patient interaction and patient satisfaction.. Pediatrics.

[PDF_00562] Kupst M. J., Dresser K., Schulman J. L., Paul M. H. (1975). Evaluation of methods to improve communication in the physician-patient relationship.. Am J Orthopsychiatry.

[PDF_00568] Molleman E., Krabbendam P. J., Annyas A. A., Koops H. S., Sleijfer D. T., Vermey A. (1984). The significance of the doctor-patient relationship in coping with cancer.. Soc Sci Med.

[PDF_00571] Mosconi P., Meyerowitz B. E., Liberati M. C., Liberati A. (1991). Disclosure of breast cancer diagnosis: patient and physician reports. GIVIO (Interdisciplinary Group for Cancer Care Evaluation, Italy). Ann Oncol.

[PDF_00573] Novack D. H., Plumer R., Smith R. L., Ochitill H., Morrow G. R., Bennett J. M. (1979). Changes in physicians' attitudes toward telling the cancer patient.. JAMA.

[PDF_00575] OKEN D. (1961). What to tell cancer patients. A study of medical attitudes.. JAMA.

[PDF_00577] Reiser S. J. (1980). Words as scalpels: transmitting evidence in the clinical dialogue.. Ann Intern Med.

[PDF_00579] Roter D. L. (1977). Patient participation in the patient-provider interaction: the effects of patient question asking on the quality of interaction, satisfaction and compliance.. Health Educ Monogr.

[PDF_00587] Thomasma D. C. (1983). Beyond medical paternalism and patient autonomy: a model of physician conscience for the physician-patient relationship.. Ann Intern Med.

[PDF_00590] Watson M., Greer S., Young J., Inayat Q., Burgess C., Robertson B. (1988). Development of a questionnaire measure of adjustment to cancer: the MAC scale.. Psychol Med.

